# From crisis to self-confidence and adaptation; Experiences of being a parent of a child with VACTERL association – A complex congenital malformation

**DOI:** 10.1371/journal.pone.0215751

**Published:** 2019-04-19

**Authors:** Ann-Marie Kassa, Helene Engstrand Lilja, Gunn Engvall

**Affiliations:** 1 Department of Women’s and Children’s Health, Uppsala University, Uppsala, Sweden; 2 Department of Pediatric Surgery, University Children's Hospital, Uppsala, Sweden; DEFACTUM, DENMARK

## Abstract

**Aim:**

Knowledge is scarce regarding mothers’ and fathers’ experiences of being a parent of a child with VACTERL association—a complex malformation. The aim of the study was to describe experiences of being a parent of a child with VACTERL association.

**Method:**

Semi-structured interviews were performed with ten mothers and nine fathers face-to-face or by telephone and analyzed by using Qualitative content analysis.

**Results:**

The parents described crisis reactions at the discovery of malformations in their child. Involvement in care was reported from the initial hospital admission until actively taking responsibility for treatments at home. Eventually the health condition became an integrated part of everyday life. The parents expressed the importance of meeting other families with a child with VACTERL. Descriptions were given of more or less professionalism with perceived discrepancies of knowledge and experience between the healthcare professionals in the tertiary hospital and those in the local hospital. Difficulties in receiving medical support during the initial period at home were described. Furthermore, emotional support and practical arrangements regarding parental accommodation and transportation varied.

**Conclusion:**

Being a parent of a child with VACTERL association involves crisis, mixed emotional reactions and shared responsibility for the child´s treatment and care with the professional care providers. Psychological processing, good medical care and support from experts, and peer support from other parents is essential in the parents’ struggle to reach self-confidence and adaptation. A care plan with individualized tailored care for each child including a training and support plan for the parents is warranted. To reduce the described discrepancies in knowledge and experience between the local and tertiary hospital, video sessions with the parents and responsible professionals at the local and tertiary hospital could be an appropriate mode of transferring information at discharge and follow up of the child.

## Introduction

VACTERL association is a complex condition of congenital malformations that coexist in a single patient and the acronym stands for vertebral defects (V), anorectal malformations (A), cardiac defects (C), tracheo-esophageal fistula (TE), renal anomalies (R) and limb abnormalities (L). At least three of these conditions need to be present for a VACTERL diagnosis [1]. For survival, most children require surgery during the first days of life and often repeatedly during childhood [1]. In spite of advanced surgery, various physical sequelae may remain lifelong. Bowel dysfunction is common in children with anorectal malformations (ARM) often requiring daily treatment with enemas. Cardiac defects may result in impaired physical capacity and children with esophageal atresia (EA) often suffer from dysphagia, gastroesophageal reflux and respiratory symptoms [1–4]. Children with VACTERL association may need extra support and adjustments in school due to physical dysfunction and attention difficulties [4].

The discovery of a congenital malformation in the fetus or new-born child induces a crisis reaction in the parents [5] with emotions of grief over the loss of an expected healthy child [6–8], shock [7], chaos, [9], sadness, anxiety [10] and worry related to the child’s health condition, treatment and the uncertain prognosis [8,11,12]. Ambivalent emotions have also been described as alternating between fear of losing the child and hope for successful treatment [10,11]. Increased psychological distress and anxiety compared to parents of healthy infants has been measured in parents shortly after the birth of their child with congenital malformation [13] and this might remain for many years [14] including acute stress disorder or posttraumatic stress disorder [8,15].

Having a child in need of care in a neonatal intensive care unit (NICU) entails stress for the parents in this environment described as an “alien world” [16]. The development of the parent-child relationship could be affected [17] by decreased possibility to hold and feed the child [8,11,18]. The parents experience a need of being close to the child and observing what is happening to gain a sense of control and a need for continuous and realistic information [16]. Mistrust towards the healthcare professionals can be experienced on account of prescriptions that have not been followed and meeting inexperienced healthcare professionals [16]. Also, during later hospital admissions, the parents need to feel secure in the perceived competence of the health professionals and to convey this security to their child by being close [19]. Parents of a child with VACTERL association are left with responsibilities for medical treatment at home particularly in children with anorectal malformations [20], performing enemas and anal dilatations [9,21].

Studies including parents’ experiences of taking care of a child with congenital malformations have often focused on a single malformation, predominantly cardiac defects. However, current knowledge is scarce regarding mothers’ and fathers’ experiences of the health condition in a child with complex congenital malformations, the health care, treatments and follow up. The information from the interviews in this study may provide new insights in how health care could be improved according to the parents’ experiences. The aim of the study was to describe experiences of being a parent of a child with VACTERL association.

## Material and methods

### Participants

Parents of children with VACTERL association aged 5–8 years, treated in our tertiary pediatric surgical center were through a mailed information letter invited for interviews. A purposive sampling was used to obtain a mixed study group. Out of 12 approached families nine agreed to participate. Additionally, one family was recruited through a national peer association for families with a child with VACTERL association and contacted by themselves the researchers by e-mail. The parents provided written consent after they had received written and oral information. The findings from the interviews of the children are published elsewhere [22]. Due to language difficulties one parent was excluded and thus 19 parents were interviewed, 10 mothers and nine fathers. The children had malformations of various locations and severity but all were diagnosed with either anorectal malformation and/or esophageal atresia and nine underwent surgery during their first few days of life. Out of the 10 children, four had undergone cardiac surgery and three spinal surgery. The births of nine of the children took place in local hospitals while one child was born in a tertiary hospital (Table 1).

**Table 1:** Characteristics of children of the interviewed parents.

Sex	Age at parent´s interview	Malformations	Discovery of malformations	Delivery site
Boy	5 y 3 m	A, C, TE	At birth	Local hospital
Boy	5 y 3 m	V, A, other*	Prenatally, at birth	Local hospital
Girl	5 y 5 m	V, A, C**	At birth	Local hospital
Boy	5 y 10 m	V, A, TE, R	At birth	Local hospital
Boy	6 y 0 m	V, A, C	At birth	Local hospital
Boy	6 y 2 m	A, C, TE	At birth	Local hospital
Girl	7 y 3 m	V, C**, TE	At birth	Local hospital
Girl	7 y 5 m	V, C, TE	At birth	Tertiary hospital
Girl	8 y 2 m	V, A, C**	At birth	Local hospital
Girl	8 y 10 m	V, A, C**	At 5,5 months age	Local hospital

**V** = Vertebral defect, **A** = Anorectal malformation, **C** = Cardiac defect, **TE** = Tracheo-esophageal fistula, **R** = Renal anomaly.

*Diagnosed as VACTERL association by pediatric geneticist

**In need of cardiac surgery.

### Method and data collection

An interview guide was constructed by the authors and used to cover the aim of the study (S1 File, S2 File). The open-ended questions concerned the parents’ experiences of being a parent of a child with VACTERL association, experiences of hospital care and healthcare support for the child at home. The parents were asked to provide suggestions for improvements in the health care. The first main questions was: *How did you experience the first period in hospital when your baby was newborn*? Follow up questions like *“Can you describe…*?*”*, *“How did you feel…*?*”* were used to deepen the answers. Between December 2015 and November 2016, the first author, an experienced pediatric nurse and PhD student who was not involved in the treatment of the children, performed the interviews. Interviews of two mothers were performed in an undisturbed room in the hospital and of one father in the family home. The remaining eight mothers and eight fathers were interviewed by telephone. Before starting the actual interview the researcher introduced herself and the aim of the study and after some small talk the voluntariness of participating was emphasized as well as the possibility to refrain from answering some questions or to terminate the interview at any time. Reflection notes were taken after the interviews. Median length of the interviews was 54 minutes (27–155), mothers’ 56 minutes (27–155) and fathers’ 45 (27–82) minutes. By using IBM SPSS Statistics software version 25, the Mann-Whitney U test was applied to compare the median length of the interviews between the two genders. The dialogues were audio recorded, listened through repeatedly and transcribed verbatim. The study was approved by the Regional Ethical Review Board in Uppsala, registration number 2015/264.

### Data analysis

Qualitative content analysis with an inductive approach [23] was used for analysis as described by Graneheim & Lundman [24]. The interviews were listened to and the transcripts read repeatedly by the first author to obtain a sense of the content. According the aim meaning units were identified, condensed and given a code. The total number of codes were approximately 1400 and the frequency of respondents in each subcategory as described by Schreier [25] is presented in supporting table (S1 Table). Subcategories and categories were formulated by the process of comparing, grouping codes and abstracting them, while keeping similarities within and disparities outside categories. NVivo 11 Pro for Windows software (QSR International Pty Ltd, Victoria, Australia) was used to organize and visualize the material. To ensure correct understanding of the content a movement between the whole content of the interviews and the identified codes was performed during the analysis process. Finally, a theme was formulated. The analysis was performed in collaboration between all of the authors until consensus was reached.

## Results

Parents’ experiences of being a parent of a child with VACTERL association are described in the categories: Becoming and being a parent of a child with a complex congenital malformation and Experiences of health care in conjunction with treatment of the child (Table 2). The content is described within ten subcategories illustrated by quotations followed by the study number of the respondents with F for father and M for mother. An overarching theme was formulated: From crisis through struggles with rays of hope, to self-confidence and adaptation. The crisis represents the shock and reactions; the struggles are all the effort needed to handle a child with malformations at the hospital and at home. The hope derives from professionalism among healthcare providers, improvements in the child’s health and shared experiences with peers until self-confidence and adaptation are reached and the health condition becomes an integrated part of life. No significant difference in length of mother’s and father’s interviews were found (*p* = 0,131). Both genders provided statement in all subcategories.

**Table 2:** Categories and subcategories describing fathers´ and mothers´ experiences of being a parent of a child with VACTERL association.

Categories	Subcategories
**Becoming and being a parent of a child with a complex congenital malformation**	Experiencing acute crisis and delayed psychological reactions
	Being involved in the child’s care from providing closeness to taking active responsibility
	Experiencing existential reflections and ambivalent emotions about procedures and complications
	Perceiving their child’s acceptance and dislike of health care in hospital and at home
	Sharing experiences with others and gaining strength to handle the situation
	Accepting and integrating the health condition into the life of both parents and children
**Experiences of health care in conjunction with treatment of the child**	Experiencing more or less professionalism from healthcare professionals
	Receiving both appropriate and inappropriate medical and practical information
	Experiencing both adequate and insufficient support
	Dealing with more or less suitable practical arrangements

### Becoming and being a parent of a child with a complex congenital malformation

Descriptions included emotional crisis, involvement in the care of the child, ambivalent emotions regarding procedures and also the children’s experiences of health care. The parents described what gave them hope and strength and that the health condition eventually became an integrated part of their family life.

#### Experiencing acute crisis and delayed psychological reactions

At the discovery of malformations in their child the parents described a state of shock and feelings of chaos, worry, sadness, absence and emptiness: *“Didn’t know what to do with myself* … *I was so worried about how things were with [the child] actually* … *I lay out on the ground in the parking lot sometimes* … *and cried* … *I was just so very worried” (8F)*. Further, they experienced anxiety and fear during their child’s first days of life due to the uncertainty of the prognosis: *“No*, *well* … *my conviction was that* … *if you have a heart disease*, *you’ll like die” (1F)*. Lack of knowledge about the diagnosis caused fear that the child would die and even a feeling of grief: *“Then I didn’t know how many hours he would survive* … *and I didn’t expect him to get through … the surgery* … *I was pretty sure that we would be parting* … *a natural sadness” (9M)*. The first strategy the parents mentioned to handle their emotions was to repress them and deal with the current acute situation. Other methods were to focus on the positive aspects and physical activity. In spite of the crisis, the parents described a sense of hope when they realized that it was possible to perform surgery, and later on when the child had recovered and the family could return home. However, they did express a sense of being different, partly caused by neighbors distancing themselves from the family and also by not knowing anyone with a similar health condition: *“You felt like the loneliest person in the whole world*, *having a little baby with a stoma* … *there was no one else we knew who had it like us or had even heard of it” (6M)*. Later psychological reactions were reported after completed vital surgery and when less focus on practical procedures was necessary. Some parents spoke of being in bad mental condition already on discharge from the hospital but for other parents reactions were delayed by up to several years and could be manifested as panic attacks or fatigue depression.

#### Being involved in the child’s care from providing closeness to taking active responsibility

During the initial period in the hospital the parents described how they tried to be as close as possible to the child by at least holding its hand. They perceived that the child could sense their closeness by improved vital signs and becoming calm. Even though parents were encouraged to hold their child, they described uncertainty regarding what they were allowed to do shortly after surgery. The fact that it was impossible to lift up and hold the child was described as a hindrance to attachment to the child. Also, there was an initial feeling of having got an illness instead of a child: *“It took quite a long time before we* … *bonded with him as our child* … *it felt* … *we said to each other* … *like we’ve got a disease* … *that’s like going to ruin* … *our family and this wasn’t how we’d thought it would be*, *that was our first thought” (2M)*. The parents described how the healthcare professionals involved them in the hospital care from the beginning by giving them some tasks in emergency situations: *“I was tasked to make sure that the oxygen tube was as close to her nose as possible so that she was oxygenated” (5F)*. However, when too much responsibility was initially given for caring procedures this was experienced as moving the focus from the child to practical issues: “*Another thing that was a bit difficult*, *was this thing with the feeding*. *It was important all the time that he got food through the syringe*, *into his mouth* … *they … wanted us to do it* … *and that was to give us better contact*, *but* … *it was mostly really hard” (2F)*. The parents spoke of how they gradually participated more actively in the medical care and could influence treatments by putting questions and making suggestions. Various levels of how much they wanted to participate practically in the treatments and care during hospital admissions were reported. A pronounced plan made by the nurse responsible concerning the division of duties between healthcare professionals and parents contributed to a feeling of safety.

The parents described several ways of preparing their child when being responsible for facilitating in conjunction with hospital visits, by talking about the hospital and informing the child of the plan for the stay. Furthermore, they reported how they isolated the family ahead of planned admissions to avoid postponed surgery due to infectious diseases. According to the parents, they strived to stay close to the child during procedures at the hospital and in the intensive care unit. To make the stay more enjoyable the parents encouraged the child to visit the play unit and bought candy or other rewards for the child.

Health care procedures performed by the parents on their child at home were described such as handling the colostomy or urine catheter, dilating the anus and giving daily enemas or intravenous antibiotics. Some procedures were easy to perform while others were considered tough and entailed a feeling of taking a great responsibility. For procedures involving the anal region some fathers did not manage to perform them since they perceived them as abuse. In addition, being challenged by other parents insinuating that these procedures were abuse was described as a hard experience: *“what’s maybe the most difficult* … *then I met someone who had children of the same age* … *you are well aware that you are abusing them every day*, *she said*. *And then … you start thinking*, *but maybe that's what I’m doing*, *but you know it's not*, *because you know it's for the good of the child” (4M)*.

#### Experiencing existential reflections and ambivalent emotions about procedures and complications

The parents shared how they struggled with the meaning and motive of having a child since it had to endure difficult procedures. Despite suffering with their child, the parents could regard every procedure as a step towards improvement in the child’s health condition. Still, to hand over their child for anesthesia and surgery always entailed a horrible feeling which never disappeared and the emotions were the same irrespective of whether it was short or long anesthesia. Worries about complications during the anesthesia were described and they were accentuated if the procedure took more time than the estimation given in advance: *“The feeling of giving your child up to anesthesia* … *it can’t be described in words*, *because it is so sad and loathsome* … *and behind it there’s such pain*. *Then I think it's okay when you know that you've got started now*, *but you never know what's going on in the meantime” (9M)*. Further, the parents expressed worries in connection with adverse events in care, such as overdosing of drugs and hospital-acquired infections.

#### Perceiving their child’s acceptance and dislike of health care in hospital and at home

The children’s perception of hospital visits were by the parents described as being a natural part of their lives and even though the children had mixed feelings they quite enjoyed them: *“he thinks it's both very hard to go to the hospital but also a little fun” (2F)*. According to the parents, positive aspects were playing, clowns, rewards and the healthcare professionals. They spoke of how the children’s dislike of hospital visits was based on feelings of uncertainty, fear or worry about meeting new healthcare professionals, people dressed in white and not knowing what would happen. According to the parents, the children were worried about anaesthesia but still preferred it to being awake during procedures. The parents stated that the children’s negative feelings were often associated with needles and that they needed information, preparation and to be involved during procedures: *“She is mostly happy and positive* … *but it's these needle sticks that have been the big dilemma” (1M)*.

The parents described the dilatation of anus performed at home as hard and painful for the child, and it sometimes resulted in aggressive behavior towards the parent: *“One of the hardest* … *you had to dilate the anus with those there Hegar dilators* … *it was no fun time*, *the morning and evening it was torture for him” (6F)*. According to the parents, it was easier for the child to tolerate this treatment during infancy compared to the age of 3–4 years when they were still unable to understand the reason. Conversely, it was described how a children aged 5 could understand the motive for enemas and the connection to their wellbeing.

#### Sharing experiences with others and gaining strength to handle the situation

To meet other families with a child with VACTERL association during visits to a national competence center for rare diseases or through the peer organization was by the parents described as beneficial. These encounters provided further recognition of the health conditions and in addition a natural connection among the children. For parents with small children the meeting with older children with the same diagnosis created hope for the prognosis of their own child: *“then we saw older children* … *then we said* … *they're just like everyone else but with their problems* … *it was nice to see that you can live a good life anyway*, *because that's what we did not know at the beginning” (2M)*. Some families considered it helpful to have a contact family including a child with a similar diagnosis. Difficulties in finding the peer organization were described and wishes conveyed that someone should invite them in and arrange membership. In addition, the parents expressed how they gained strength to handle the situation by experiencing the child’s wellbeing, development and positive attitude and through the relations within the family.

#### Accepting and integrating the health condition into the life of both parents and children

The parents expressed that the health condition of the child and its procedures eventually became incorporated in everyday life. Without thinking much about the condition they were prepared for new health problems to come: *“Now A [the child]*, *he’s A and not his illness*, *but we don’t think so much about the disease* … *it was much more so when he was little* … *now you don’t feel that you need to* … *we simply don’t think so much about it” (2M)*. According to the parents, the health condition had become an integrated part of life for the children as well since they knew of nothing else. Parents described that even though the children had been teased periodically, they were positive, had friends and could enjoy talking about their medical history.

### Experiences of health care in conjunction with treatment of the child

The parents described experiences of varying degrees of professionalism and of communicated information from the healthcare professionals. The parents provided descriptions of medical care and social support in the hospital and various levels of medical and practical support at home. Furthermore, practical arrangements for parental accommodation and transportations were described.

#### Experiencing more or less professionalism from healthcare professionals

The parents experienced commitment and respect from the healthcare professionals in the hospital. However, they also gave examples of healthcare professionals behaving disrespectfully or leaving the parents out when discussing the child’s condition: *“And then she [the doctor] comes back* … *and so she says [to the nurse]* … *as you have heard*, *the operation is canceled* … *I understand that it’s hellishly wrong*, *my daughter’s going to die*, *there is* … *no point in operating on a child that’s going to die so it is clear she’s going to die” (1M)*. The level of dedication to their work was apparent to the parents who nevertheless described very committed healthcare professionals but also those showing a lack of responsibility by neglecting symptoms, questioning treatments or trying to disparage mistakes in treatment. The parents spoke of sensitivity on the part of the healthcare professionals as when they as parents felt they were being listened to and considered to be the experts on their children. The opposite was also described when the parents’ worries and wishes were not taken seriously and their reports about their children were neglected. Furthermore, they expressed the importance of the health professionals being calm, since stress among them was perceived, spread to the parents and reduced the possibilities to talk to the healthcare professionals.

The healthcare professionals were described as knowledgeable, competent and professional by some parents. Other parents expressed a lack of confidence in the healthcare professionals’ ability to handle acute situations, both in the neonatal unit and in the pediatric ward. In addition, healthcare professionals performing care actions that were perceived as contradictory to prescriptions triggered anxiety. Moreover, the parents perceived limited knowledge and experience of the diagnosis in the local hospitals. According to the parents, routines were lacking in the delivery room regarding what actions to take when a child was born with a malformation. For example, a nurse tried to create an opening on the site of anus in a child with anorectal malformation: *“They didn’t know themselves* … *it was some nurse who was going to make a hole*, *there was no anal opening* … *so she stood there with the thermometer and tried” (6F)*. In general, the parents experienced child competence among healthcare professionals as being when the children were treated with respect through direct communication before talking to the parents. In addition, the healthcare professionals used various methods for preparing for procedures such as combining information with books, dolls and playing. Conversely, a lack of child competence was reported in interactions with healthcare professionals in primary care contexts.

#### Receiving both appropriate and inappropriate medical and practical information

According to the parents, the content of the communicated medical information about the diagnosis, prognosis and treatment was good, and was given orally and rarely in writing. However, initial information from the local hospital was experienced as incorrect and simplified: *“Then someone turned to us and said like*, *maybe you may have to go to [the tertiary hospital] and just make a hole* … *and then it will be fine again” (6M)*. According to the parents it was difficult to understand and assimilate the information due to worries and uncertainty regarding the child’s prognosis. When a physician exclusively addressed the information to just one of the parents though both were present, a feeling of not being included was experienced. The parents spoke of good methodical information such as being continually updated, receiving explanations, being objective and honest and detailed, and providing possibilities to pose questions. In addition, they wished that this should be realistic and include information about possible complications. A less appropriate method of communicating information was experienced when the physician described the surgical details before saying if the child had survived: *“*… *so he began to draw on a piece of paper and explained that* … *[the child] was born with a heart that looked like this* … *he never said if she was alive*. *And that's what we wanted to hear” (5M)*.

Concerning the methods of information, parents wished for more information opportunities with physicians and specialist nurses, allowing for further direct follow-up conversations with the nurses. Moreover, the parents asked for an assigned contact person to receive information from during the whole process. Written information was requested about the diagnosis but also after every hospital visit, describing examinations, results and plans. Another concrete suggestion was to use a “care book” for writing down information about all healthcare contacts. According to the parents, good practical information was given about plans for an admission period and available service in the hospital ward. However, even more information was requested e.g. before transferal to another ward and also about available assistance with minor practical issues. Further, the parents asked for more information concerning planned procedures ahead of short hospital visits, available aids for the child, financial grants and peer organizations.

#### Experiencing both adequate and insufficient support

Parents described how they felt secure with the good medical care provided in the hospital since the life of the child was saved and all medical disorders were checked. The whole family was supported and was well taken care of: *“I thought they spent as much time with the parents as with children who were in the hospital” (10M)*. After discharge from the hospital some parents described good accessibility to healthcare professionals. A physician personally responsible for their child provided support at the local hospital. Furthermore, easily accessible support from the physician in the tertiary hospital was experienced as providing the possibility to solve acute situations through telephone contact. The parents described the support from nurse specialists, particularly bowel therapists, as valuable with initially frequent contacts, commitment and the possibility to receive advice by phone: *“These contacts you've got are important* … *with the bowel and stoma nurses* … *you can talk to them peacefully and calmly and you can feel you trust them*, *these contacts have been very important” (8M)*. Conversely, some parents described difficulties during the initial period at home in getting in touch with the local hospital, delays in getting a personal physician with responsibility for their child and overall support for the various health problems of the child. Likewise, when it came to practical support at home, parents described a feeling of initially being left alone without knowing who to contact when needing assistance in e.g. treatments, breast feeding and care of colostomy: “*we had learned about stoma but we were very insecure* … *but when we got there [local hospital] the healthcare professionals said* … *we have heard that you can do this so well that we haven’t taken the time to* … *we haven’t learned it” (6M)*. Furthermore, difficulties in getting aids and support from the occupational therapist were described by some, while other parents described sufficient practical care support provided by the child health center and in the supply of necessary bandages or incontinence aids.

Some parents described how the tertiary hospital provided functioning coordination for the follow up of the child. However, other parents missed a care plan at discharge and coordination between the local and tertiary hospital. These parents described frustration over having to coordinate and chase the various contacts and sometimes being forced to nag to receive relevant follow up. Wishes were expressed for a person coordinating contacts and performing long-term planning concerning the different hospitals treating the child.

Some parents stated that they received valuable emotional support through counseling with a social worker, psychologist or deacon at the hospital. Even so, dissatisfaction was expressed with the content focusing on practical issues rather than on the core experience of having a sick child. Other parents stated that they were either not offered or declined the offer since they were not interested or considered the daily practical issues more important than counseling. Suggestions were made that counseling should be routine during the first hospital stay and that the offer should be repeated. Parents expressed discontent at not receiving information about and assistance to apply for financial support but also reported how they themselves postponed application of care allowances since it was too tough to describe the everyday difficulties.

#### Dealing with more or less suitable practical arrangements

The parents described how at least one parent could stay close to the child also during the night in the neonatal ward or in a parents’ room and how efforts were made to arrange for both parents to be close to the ward. Staying in the parents’ quarters was considered appropriate with possibilities to meet other parents in similar situation. Less appropriate settings were the maternity ward and hotels where the parents were surrounded by happy people while they themselves were sad and doubtful of their child’s survival: *“then we are convinced that we have a daughter who’s going to die during the night* … *and then we're going to like be sleeping in the maternity ward*, *with all these happy mothers running around with their little children* … *so it was really tough* … *it was almost malicious” (1F)*. When the child was older it was always possible for one parent to stay with the child in the hospital room except for when being cared for in the intensive care unit. It was a clear desire that both parents should have this opportunity. The parents perceived the hospital wards as stressful environments with shortages of beds resulting in early discharge. Furthermore, the parents described troublesome transportations between the airport and the hospital, in big cities or on long journeys for daily treatments. When the child was transferred to the tertiary hospital by an emergency flight, transportation was not always arranged for the parents and it was reported how they drove a long distance in their own car shortly after delivery.

## Discussion

In the present study we have investigated experiences of being a parent of a child with VACTERL association. The parents described crisis reactions at the discovery of the malformations in their child. Gradually they actively took increasing responsibility for treatments of the child and performed procedures at home which were sometimes considered as demanding. Through medical care and support, support from peers and family, and noticing the child’s well-being and development, the parents could perceive hope. Eventually the health condition became an integrated part of everyday life. Descriptions were given of greater or less professionalism in healthcare professionals and difficulties in receiving medical support during the initial period at home. Furthermore, the parents described various extents of emotional support and practical arrangements regarding parental accommodation and transportation.

The parents in the present study described similar reactions to those earlier reported fearing that their child would die regardless of whether he/she was born with cardiac or other not immediate life-threatening malformations [5,8,11]. It is imperative to recognize each parent’s individual needs, meet the parents with sensitivity and to provide repeated, realistic and honest as well as optimistic information about the condition and the expected prognosis. Furthermore, parents described how anesthesia brought on anxiety for complications despite previous positive experiences. To avoid an increase in the parents’ anxiety when the surgery is protracted [26] it is necessary to provide a realistic estimation of the duration of the procedure. Moreover, after surgery, it is appropriate to inform about the current condition of the child before describing details. The need of psychosocial support was expressed by the parents even though they had not considered it necessary during the initial hospital stay. Therefore, it is essential to offer repeated opportunities to visit a psychologist as a routine in the care plan, to support the processing of the traumatic event of having a child with malformation. The parents described how they felt lonely in the new situation without the acquaintance of a child with similar condition, as also described by other researchers [21]. Benefits from meeting other children with the same diagnosis and their parents were expressed. Sharing experiences provided them with recognition and hope for the future and it has been reported how these encounters can result in mutual support and sometimes long lasting relations [27]. Thus, it is important to provide the parents with information of a contact family and an invitation to a diagnosis specific peer association as soon as possible so they can make an appointment when it is suitable from their point of view. Practical solutions are required regarding the described needs of the parents. When arranging accommodation during the initial admission it is essential to consider the parents’ fear of losing their child and avoid mixing them with happy parents and newborn healthy babies. Rooms in parental quarters intended for parents of sick infants should be the first choice, and this might also provide contacts with other parents of children with similar conditions.

The parents described how they were initially involved in the care by being close to the child and that they were given some duties already in the acute situation. The importance for both the child and the parents of parental involvement in the Pediatric Intensive Care Unit have been previously highlighted [26]. According the present study, sensitivity to the parents’ wishes and capability is required in this situation. Therefore, it is important to prevent too much responsibility from the beginning as this can risk altering the focus from bonding with the child to only practical duties. After discharge the parents were given responsibility to perform medical procedures especially in the children born with anorectal malformations. Dilatations of the new constructed anal opening are recommended twice per day in children reconstructed with posterior sagittal ano-rectoplasty to prevent strictures [28] and are generally used as a routine in centers specialized in colorectal surgery [29]. For prevention and treatment of constipation and fecal soiling enemas might be needed daily [28]. In particular, the dilatations were described by the parents as painful and by some fathers they were considered as abuse of the child. Other parents have also described the procedure as troublesome [20,21] and complications such as pain and bleedings have been reported [30]. Experiences of long-term dilatations performed by parents during childhood have been correlated to increased psychological dysfunction in adolescence and adulthood [31]. In addition, the procedures could incur parental stress and affect the child-parent relationship [31]. Hence, more research including large cohorts is required to evaluate the evidence of the treatment and to identify methods to alleviate the unpleasant experiences for children of any age. Thus, parents need knowledge and guidance in performing specialized treatment. A care plan with individualized tailored care for each child and for the training and support of parents is warranted. The stoma therapists play a significant role in supporting the parents and providing practical advice on how to make the procedure as gentle as possible.

The parents described how they through their experiences became the experts regarding their child’s health condition. They expressed self-confidence and the ability to influence the treatment and described how they eventually became adapted to the health situation as an integrated part of life. It has been suggested that active participation in the treatment of the child could prevent the risk of posttraumatic stress disorder in mothers of children born with malformations [15]. The parents in the present study might have developed in their parental role through the hardships they have overcome and through coping with the situation as described in parents of children with anorectal malformations [9]. Similar processes may also explain why parental stress in parents of older children born with malformations was similar or lower compared to a national sample [9,32].

According to the parents, routines for adequate actions when the child was born with a malformation did not exist in the delivery room. This is in line with findings from other researchers describing parents’ experiences of insecurity among healthcare professionals in similar situations [21]. Therefore, a manual of how to handle various unexpected malformations should be developed and provided together with training to improve the routines. Even though some parents reported good support from the local and tertiary hospital directly after discharge other parents described a lack of necessary assistance. There seems to be a discrepancy in knowledge between the tertiary and the local hospital due to scarce experience of the diagnosis in the local hospital. To transfer knowledge to the local hospital, video sessions could be implemented at the discharge of the child and at follow up. For the family, one great advantage would be less traveling to the tertiary hospital. In addition, the video sessions would provide assurance to the parents by perceiving the exchange of information and agreement of treatment between the tertiary and the local health professionals. VACTERL association often entails various sequelae [2,3] and difficulties in daily functions [4] with a need for lifelong follow up. The parents described how they had to keep track of all the different healthcare contacts. As suggested in the present study, it would be worthwhile for parents of children with VACTERL association to have access to one coordinator with an overview of the planning for all the child’s healthcare contacts.

Both mothers and fathers provided statements in all subcategories. This could indicate that both parents were engaged in being a parent of a child with malformations and that they actively took part in the treatments and procedures. This is possible around the birth of a child with a malformation because one parent has parental leave and the other parent has access to sick leave in accordance with the Swedish social welfare system. Furthermore, parents may continue to share responsibility during the child’s upbringing. Several similarities were found between the statements of the parents in the present study and their previously interviewed children [22]. Both the children [22] and their parents described the children as being happy and reported both positive and negative experiences of hospital visits. Experiences of dilatations were described by some parents while only one child mentioned them and the associated pain [22]. This could be explained by the fact that most children undergo dilatations during infancy and only a few at older ages.

### Methodological considerations

To our knowledge this was the first interview study reporting on experiences of being a parent of a child with VACTERL association. During the analysis process the interviews were read repeatedly and the constructed codes checked continually with the transcribed interview. To additionally increase credibility of the analysis, considerations and discussions of the content of the categories and subcategories were performed repeatedly among the authors until consensus was reached. An overarching theme was formulated describing the underlying content and the process that the parents undergo [33].

A strength of the study was that the interviews were performed by the first author who was not involved in the regular care of these children, which may have contributed to the parents freely sharing their experiences which in turn resulted in rich data. In addition, disparity as well as similar aspects were described within subcategories, supporting good saturation of data. Furthermore, credibility was enhanced owing to both mothers and fathers being interviewed [24]. The content was covered by both mothers and fathers providing statements in all subcategories, thus contributing data from both genders. The number of respondents per subcategory show occurrence of statements and are not a quantitative measure when using qualitative method with a small sample. Further studies could explore psychosocial aspects from mothers and fathers, such as involvement in the child´s care, emotions and coping, and the process of acceptance and integration of the child´s health condition in the daily life over time, using qualitative and/or quantitative methods.

The telephone was used in 16 of the 19 interviews. Based on the impressions from the interviews, the parents in this interview setting vividly shared their experiences. Even though the wordless communication between the interviewer and informant is lost, telephone interviews can yield information of similar amount and quality as face-to-face interviews [34]. Furthermore, the informants may be more relaxed, finding it easier to discuss sensitive subjects [35–37], could be more honest [36] and not least appreciative of the more practical way to participate [34,36].

A limitation of the study was the small study group including 19 parents of 10 children with a rare diagnosis which might reduce the transferability of the findings to other groups and contexts. However, our findings were confirmed by the similar reactions of the parents’ to the diagnosis of their child [5–12], experiences during initial hospitalization [11,12,16–18] and of taking responsibility for treatments [20,21], comprising various diagnoses. These experiences might be common to parents of children diagnosed with various congenital malformations or other chronic health conditions, which strengthens the transferability to similar contexts [24].

## Conclusions

Being a parent of a child with VACTERL association involves crisis, mixed emotional reactions and shared responsibility for the child’s treatment and care. A child’s complex malformation often entails long time follow up, repeated episodes of anesthesia and surgery and difficulties in everyday functions. Psychological processing, good medical care and support from experts and other parents is essential in the parents’ struggle to reach self-confidence and adaptation. A care plan with individualized tailored care for each child including a training and support plan for the parents is warranted. To reduce the discrepancies in knowledge and experiences described between the local and tertiary hospital, video sessions with the parents and responsible professionals at the local and tertiary hospital could be an appropriate mode of transferring information at discharge and follow up of the child.

## Supporting information

S1 File Interview guide in original language (Swedish). (DOCX)

S2 File Interview guide translated into English. (DOCX)

S3 File COREQ checklist. (DOCX)

S1 Table Categories, subcategories and respondent counts. Fathers (F) and mothers (M).
(DOCX)
