# Supplementary material for: From crisis to self-confidence and adaptation; Experiences of being a parent of a child with VACTERL association – A complex congenital malformation
Source: PLoS One. 2019 Apr 19;14(4):e0215751. doi: 10.1371/journal.pone.0215751 (PMC6474607; doi:10.1371/journal.pone.0215751)
Supplement: S1 File — (DOCX) [file pone.0215751.s001.docx]

S1 File. Interview guide in original language (Swedish).

# Intervjuguide föräldrar till barn 5- 8 år

Presentation av mig själv, varför intresserad.

Syfte med intervjun: Upplevelser av vården av ditt barn

Hur går det till?

Berätta gärna fritt! Inga svar är rätt eller fel.

Jag spelar in - skriver ner intervju kodat

Anonym sammanställning av resultatet. Ingen vet vad just du svarat.

Kodlista som enbart jag och mina handledare har tillgång till.

Frivilligt!

**Hur var det innan ert barn föddes:** Vad visste ni? Kunde ni förbereda er?

**Hur var det när han/hon föddes? Var? Vad hände?**

**Hur var första tiden på sjukhus när ert barn var nyfött?**

Hur var tiden på sjukhuset?

Hur var informationen?

Hur har barnet bemötts?

Hur har du som förälder blivit bemött?

Hur delaktiga i planeringen har ni fått vara

Är det något du saknat?

Vad önskar du hade varit annorlunda?

**Hur var tiden på sjukhus vid senare vårdtillfällen: Vilka sjukhus? Flera avdelningar på specialistsjukhuset?**

(Upprepning av frågor ovan)

**Hur har kontakten med sjukvården varit sedan ni kom hem?**

Tillgänglighet? Hur har det varit att få kontakt vid behov?

Hur har stödet varit?

Hur delaktiga har ni fått vara?

Hur har informationen varit?

Har ni saknat något?

**Vad har** **varit bra i kontakten med vården?** Berätta mer om det

**Vad har varit sämre eller rentav dåligt.** Berätta om det.

**Har du förslag till förbättringar? Vad önskar ni varit annorlunda?**

**Hur uppfattar du att ditt barn har upplevt kontakterna med sjukvården?**

När du tänker på helheten - All kontakt med sjukvården sedan ditt barn föddes - Vad tänker du då? - Vad känner du då?

**Vad har hjälpt och styrkt dig att hantera den här situationen?**

**Är det något mer du vill berätta?**

**Sammanfattning hur jag uppfattat informationen i intervjun**

**Får jag återkomma om jag har frågor?**

**Uppföljningsfrågor:** Hur upplevde du det?

Hur menar du då?

Kan du beskriva…..? Kan du berätta…….? Hur kändes det då….?

Vad gjorde du då? Kan du säga något mer om det?
