# Supplementary material for: From crisis to self-confidence and adaptation; Experiences of being a parent of a child with VACTERL association – A complex congenital malformation
Source: PLoS One. 2019 Apr 19;14(4):e0215751. doi: 10.1371/journal.pone.0215751 (PMC6474607; doi:10.1371/journal.pone.0215751)
Supplement: S2 File — (DOCX) [file pone.0215751.s002.docx]

S2 File. Interview guide translated into English

Interview Guide for Parents of Children 5-8 Years of Age

Presentation of myself, why interested.

Purpose of the interview: Experiences of the care of your child

How does it take place/How is it done?

Please speak freely! There are no right or wrong answers.

I record the interview - transcribe it, code by numbers

Anonymous compilation of the result. No one knows what just you have answered.

Code list that only I and my supervisors have access to.

Voluntary!

**How was it before your child was born:** What did you know? Could you prepare yourself?

**How was it when he/she was born? Where? What happened?**

**How did you experience the first period in hospital when your baby was newborn?**

How was your time in the hospital?

How was the information?

How has your child been treated/taken care of?

How have you been treated/taken care of as a parent?

How much have you been allowed to be involved in the planning?

Is there anything you felt you lacked?

What do you wish had been different?

**How did you experience your subsequent visits to hospitals?**

**Which hospitals? More departments at the specialist hospital?**

(Repetition of questions above)

**How has the contact with the healthcare services been since you came home?**

Accessibility? How has it been to get in touch when necessary?

How has the support been?

How involved have you been allowed to be?

How has the information been?

Have you lacked anything?

**What has been good in your contact with the healthcare services?** Tell me more about it

**What has been poorer or even bad?** Tell me about it.

**Do you have any suggestions for improvements? What do you wish had been different?**

**How do you perceive that your child has experienced the contacts with the healthcare services?**

When you think about it on the whole - All your contacts with the healthcare services since your child was born - What do you think about it? What do you feel about it?

**What has helped and strengthened you in dealing with this situation?**

**Is there anything more you would like to tell me?**

**Summary of how I perceived the information in the interview.**

**Can I get back to you if I have questions?**

**Follow-up questions:** How did you experience it?

How do you mean?

Can you describe...?

Can you tell me…?

How did you feel then…?

What did you do then?

Can you say anything more about it?
