# Supplementary material for: From crisis to self-confidence and adaptation; Experiences of being a parent of a child with VACTERL association – A complex congenital malformation
Source: PLoS One. 2019 Apr 19;14(4):e0215751. doi: 10.1371/journal.pone.0215751 (PMC6474607; doi:10.1371/journal.pone.0215751)
Supplement: S3 File — (DOCX) [file pone.0215751.s003.docx]

S3 File. COREQ checklist.Consolidated criteria for reporting qualitative studies (COREQ): 32-item checklist

| **No** | **Item** | **Guide questions/description** |
| --- | --- | --- |
| **Domain 1: Research team and reflexivity** |  |  |
| Personal Characteristics |  |  |
| 1. | Interviewer/facilitator | Which author/s conducted the interview or focus group?  *The first author (A-MK) conducted the interviews, reported in Material and Methods.* |
| 2. | Credentials | What were the researcher's credentials?  *1:st author: PhD student, 2:nd author: PhD, MD, 3:rd author PhD, see the affiliations.* |
| 3. | Occupation | What was their occupation at the time of the study?  *1:st author: Pediatric nurse, research nurse, 2:nd author: Pediatric surgeon, associate professor, 3:rd author: Pediatric nurse, senior lecturer.* |
| 4. | Gender | Was the researcher male or female?  *All authors female.* |
| 5. | Experience and training | What experience or training did the researcher have?  *1:st author: More than 10 years experience as in-patient pediatric nurse, trained as pediatric and research nurse, post-graduate courses in Interview techniques in* *health and care and in Qualitative content analysi*s.  *Short description included in Material and Methods; Method and data collection.* |
| Relationship with participants |  |  |
| 6. | Relationship established | Was a relationship established prior to study commencement?  *The first author was not involved in the regular care of these children. Relationship was established through telephone contact after an information letter was sent and consent obtained. Approximately half of the parents had met the first author at some occasion before the interview in connection with research studies, reported in the Material and Methods; Method and data collection.* |
| 7. | Participant knowledge of the interviewer | What did the participants know about the researcher?  *The invitation letter included information about the 1:st author being an experienced pediatric nurse involved in a PhD student project with the aim of investigating the parent’s experiences of health care provided for their children. Procedure approved by the Regional Ethical Review Board in Uppsala.* |
| 8. | Interviewer characteristics | What characteristics were reported about the interviewer/facilitator?  *The interviewer was not involved in the regular care of this group of children, reported in Material and Methods and in Discussion; Methodological considerations.* |
| **Domain 2: study design** |  |  |
| Theoretical framework |  |  |
| 9. | Methodological orientation and Theory | What methodological orientation was stated to underpin the study?  *Methodical orientation was Qualitative content analysis, reported in Material and Methods; Data Analysis.* |
| Participant selection |  |  |
| 10. | Sampling | How were participants selected?  *Parents of children with VACTERL association aged 5 – 8 years treated in our pediatric surgical center were invited for interviews. A purposive sampling was used to obtain a mixed study group. Out of 12 approached families nine agreed to participate. One additional family was recruited through a national peer association. Reported in the Material and Methods; Participants.* |
| 11. | Method of approach | How were participants approached?  *The families were approached by mail and telephone contact after consent obtained. Through the peer association information was spread in their web site and in meetings, reported in the Material and Methods; Participants.* |
| 12. | Sample size | How many participants were in the study?  *19 parents of totally 10 children, whereof 10 mothers and 9 fathers, reported in the Material and Methods; Participants.* |
| 13. | Non-participation | How many people refused to participate or dropped out? Reasons?  *Out of 12 approached families nine provided consent. Thus, three families did not agree to participate. One additional family contacted by themselves the researcher. One father was not interviewed due to language difficulties. Reported in the Material and Methods; Participants.* |
| Setting |  |  |
| 14. | Setting of data collection | Where was the data collected?  *Two mothers were interviewed in an undisturbed room in the hospital during planned visit, one father was interviewed in his home and the remaining participants were interviewed by telephone in their homes, reported in the Material and Methods; Method and data collection.* |
| 15. | Presence of non-participants | Was anyone else present besides the participants and researchers?  *In two cases their interviewed child were present in the same room occupied with other activities.* |
| 16. | Description of sample | What are the important characteristics of the sample?  *The interviewed parents were 10 mothers and nine fathers. Out of their 10 children nine were delivered in local hospitals, and one in the tertiary center. In nine children the malformations were discovered at delivery and they underwent surgery during their first few days of life. A table is included in the manuscript containing the children’s sex, age at the time of the interview, malformations and time of discovery and delivery site. Interviews were performed between December 2015 and November 2016, Reported in the Material and Methods; Method and data collection.* |
| Data collection |  |  |
| 17. | Interview guide | Were questions, prompts, guides provided by the authors? Was it pilot tested?  *Interview guide constructed in close cooperation with the supervisor. No pilot testing performed. Interview guide in original and English language added as Supporting information (S1 File, S2 File).* |
| 18. | Repeat interviews | Were repeat interviews carried out? If yes, how many?  *No repeated interviews were carried out.* |
| 19. | Audio/visual recording | Did the research use audio or visual recording to collect the data?  *Audio recording performed, reported in the Material and Methods; Method and data collection.* |
| 20. | Field notes | Were field notes made during and/or after the interview or focus group?  *Reflections after the interviews were written down, reported in the Material and Methods; Method and data collection.* |
| 21. | Duration | What was the duration of the interviews or focus group?  *Median length 54 minutes (27-155), mothers’ 56 minutes (27-155) and fathers’ 45 (27-82) minutes, reported in the Material and Methods; Method and data collection.* |
| 22. | Data saturation | Was data saturation discussed?  *Yes, data richness and saturation was included in the Discussion.* |
| 23. | Transcripts returned | Were transcripts returned to participants for comment and/or correction?  *Transcripts were not returned.* |
| **Domain 3: analysis and findings**z |  |  |
| Data analysis |  |  |
| 24. | Number of data coders | How many data coders coded the data?  *The analysis was performed in collaboration between the authors until consensus was reached, reported in the Material and Methods; Data analysis and discussed in the Discussion.* |
| 25. | Description of the coding tree | Did authors provide a description of the coding tree?  *The coding three is not presented in the study however, described in the text in the Material and Methods; Data analysis. A coding tree was used during the analysis by NVivo, reported in the Material and Methods; Data analysis.* |
| 26. | Derivation of themes | Were themes identified in advance or derived from the data?  *Themes were formulated from the collected data using inductive method, reported in the Material and Methods; Data analysis.* |
| 27. | Software | What software, if applicable, was used to manage the data?  *NVivo 11 Pro for Windows software (QSR International Pty Ltd, Victoria, Australia) was used for organizing and visualizing the data, reported in the Material and Methods; Data analysis.* |
| 28. | Participant checking | Did participants provide feedback on the findings?  *No participant checking was used.* |
| Reporting |  |  |
| 29. | Quotations presented | Were participant quotations presented to illustrate the themes / findings? Was each quotation identified?  *Quotations with participant number and sex illustrate the categories, reported in Results.* |
| 30. | Data and findings consistent | Was there consistency between the data presented and the findings?  *Yes, see the Results and discussed in the Discussion.* |
| 31. | Clarity of major themes | Were major themes clearly presented in the findings?  *Yes, a table is displayed for Categories and Subcategories. An overarching theme was formulated catching the underling meaning based on the findings. Reported in Results.* |
| 32. | Clarity of minor themes | Is there a description of diverse cases or discussion of minor themes?  *Yes, in the Results and in the Discussion. The finding based on the content is described in the Subcategories and quotation is added to support the labeling of the Subcategories in the Results. Supporting Information Table 1 (S1 Table) is added containing frequency of fathers’ and mothers’ contribution to each subcategory.* |
