# Supplementary material for: From crisis to self-confidence and adaptation; Experiences of being a parent of a child with VACTERL association – A complex congenital malformation
Source: PLoS One. 2019 Apr 19;14(4):e0215751. doi: 10.1371/journal.pone.0215751 (PMC6474607; doi:10.1371/journal.pone.0215751)
Supplement: S1 Table — Fathers (F) and mothers (M). (DOCX) [file pone.0215751.s004.docx]

# S1 Table. Categories and subcategories with number of respondents of fathers (F) and mothers (M).

| **Categories** | **Subcategories** | **F**  n=9 | **M**  n=10 |
| --- | --- | --- | --- |
| 1. Becoming and being a parent of a child with a complex congenital malformation | 1.1 Experiencing acute crisis and delayed psychological reactions | 7 | 9 |
|  | 1.2 Being involved in the child’s care from providing closeness to taking active responsibility | 9 | 10 |
|  | 1.3 Experiencing existential reflections and ambivalent emotions about procedures and complications | 4 | 5 |
|  | 1.4 Perceiving their child’s acceptance and dislike of health care in hospital and at home | 9 | 10 |
|  | 1.5 Sharing experiences with others and gaining strength to handle the situation | 4 | 9 |
|  | 1.6 Accepting and integrating the health condition into the life of both parents and children | 2 | 5 |
| **2. Experiences of health care in conjunction with treatment of the child** | 2.1 Experiencing more or less professionalism from healthcare professionals | 9 | 10 |
|  | 2.2 Receiving both appropriate and inappropriate medical and practical information | 9 | 10 |
|  | 2.3 Experiencing both adequate and insufficient support | 9 | 10 |
|  | 2.4 Dealing with more or less suitable practical arrangements | 9 | 10 |
